# Pessimism or optimism? A tri-spatial analysis of public risk perception bias and its influencing mechanisms: evidence from 31 major cities in China

**DOI:** 10.3389/fpubh.2026.1705187

**Published:** 2026-02-16

**Authors:** Li Wang, Yiming Wang, Minchun Wu

**Affiliations:** School of Public Policy and Management, China University of Mining and Technology, Xuzhou, China

**Keywords:** China, COVID-19 pandemic, pessimism bias, public risk perception bias, tri-spatial framework

## Abstract

**Background:**

Public risk perception bias (PRPB) is a critical psychological and social determinant that substantially shapes public health outcomes and the effectiveness of safety governance—particularly during large-scale crises like the COVID-19 pandemic. Amid increasingly complex urban risk landscapes, traditional single/dual-spatial frameworks fail to capture PRPB’s multi-source and interactive nature.

**Methods:**

This study develops an Extended Tri-Spatial Prism Model to measure PRPB across 31 major Chinese cities (2017–2023) using multi-source data and explores its spatiotemporal dynamics and multi-level influencing mechanisms via a mixed-methods approach (Delphi technique, entropy weighting, GIS spatial analysis, multiple regression, mediation analysis).

**Results:**

(1) PRPB is dominated by pessimism with marked tri-spatial heterogeneity—physical and cyber spaces show pronounced pessimism, while social space remains cognitively neutral; (2) PRPB exhibits a “north-optimistic, south-pessimistic” spatial pattern and a COVID-19-disrupted V-shaped temporal trend (2017–2023); (3) Individual and urban factors shape PRPB through the mediation of psychological stress, social capital, media rationality, and government trust.

**Conclusion:**

This study advances the theoretical understanding of PRPB by systematically delineating its tri-spatial characteristics and underlying mechanisms. These findings provide theoretical insights and practical guidance for optimizing public risk communication systems and urban safety governance frameworks.

## 1 Introduction

Public risk perception bias (PRPB) refers to the systematic discrepancy between the public’s subjective risk perception and actual objective risk. While actual objective risk can be precisely quantified via scientific methodologies and empirical data, subjective risk perception constitutes a multidimensional theoretical construct shaped by the interplay of individual characteristics, psychological traits, and sociocultural factors (1). Rooted in cognitive biases—such as probability neglect, the availability heuristic, and group polarization—PRPB primarily manifests in two forms: pessimism bias and optimism bias. Specifically, pessimism bias (overestimation bias) refers to the cognitive tendency for individuals to overestimate the probability of adverse risk-related events or underestimate the likelihood of positive events. Conversely, optimism bias (underestimation bias) denotes the opposite cognitive inclination: underestimating the likelihood of adverse risk-related events or overestimating the probability of positive events (2). In high-uncertainty risk contexts like the COVID-19 pandemic, such biases not only impair individuals’ rational judgment and trigger irrational behaviors (e.g., excessive risk avoidance or herd behavior) but also, through the “social amplification of risk framework,” generate societal consequences that far exceed the impacts of the original risk event itself (3). These far-reaching effects underscore the critical need to design targeted intervention policies for risk communication and governance.

With the evolution of post-industrial society, the universal interconnection and in-depth integration of the physical and virtual realms have emerged as defining features of modern life, driving human society toward an irreversible transition into a “risk society” characterized by high uncertainty and complexity (4). Concurrently, advances in information technology have propelled the boundaries of human activities beyond the constraints of physical space, with social space and cyber space increasingly evolving into indispensable spatial components that collectively shape a “tri-space” (5, 6). Within this tri-space framework, public safety risks—once confined to isolated physical or social spaces—have evolved into a sophisticated system shaped by the dynamic interactions across the “Cyber-Physical-Social” domains (7). Consequently, conventional single-spatial research frameworks fail to capture the multi-spatial dynamism of public safety risks in modern society. Thus, it is imperative to develop an integrated tri-spatial framework capable of explaining the generation mechanisms, evolutionary patterns, and transmission pathways of contemporary public safety risks. This framework not only addresses the spatial limitations of existing approaches but also clarifies how multi-level factors influence PRPB (including optimism and pessimism), while laying a groundwork for collaborative risk communication and governance policies that incorporate spatial and socio-psychological mechanisms.

Despite the scholarly consensus that the integration of physical, social, and cyber spaces defines modern risk contexts, existing studies on PRPB remain confined primarily to single-spatial dimensions—particularly physical or social spaces. At the physical space level, research primarily focuses on natural disasters (e.g., floods, typhoons), analyzing their risk perception patterns and behavioral responses (8–10). At the social space level, studies explore the causes and consequences of cognitive differences across social groups: for instance, Webster et al. (11) and Saleh et al. (12) identified limited specialized knowledge as a key driver of public pessimism of food safety risks and chemical risks, respectively; Măirean et al. (13) examined the links between PRPB and risky driving behaviors, emphasizing the impact of subjective risk perception on risk-related decisions. While these studies yield valuable insights, they are constrained by isolated spatial perspectives or single-type risk analyses. Specifically, they fail to adequately explore cross-spatial interaction effects and lack in-depth discussions of heterogeneous PRPB forms.

Research on the mechanisms underlying PRPB has expanded to cover micro- and meso-level analyses. At the micro level, studies focus on individual cognitive biases and their psychological mechanisms: Douglas and Wildavsky (14) applied risk culture theory to reveal group-based differences in risk perception; Siegrist et al. (15) identified food disgust sensitivity as an affective influencer of food safety risk judgments; Ando et al. (16) highlighted the moderating roles of gender and media exposure in shaping COVID-19 risk perception; and Qiu et al. (17) used the “anchoring effect” to explain workers’ recognition bias in safety evaluations. At the meso level, research emphasizes the media’s role in shaping public risk perceptions: Zhao et al. (18) conducted a natural experiment to examine the effects of disaster reporting on public compliance with tornado warnings; Tsoy et al. (19) analyzed social media’s risk-amplifying role during the COVID-19 pandemic. However, existing research suffers from a pronounced micro–meso divide—it lacks a systematic framework that integrates factors spanning individuals, communities, social media, and government, which is essential for unpacking the complex socio-psychological mechanisms of PRPB.

To address these research gaps, this study leverages integrated objective risk indicators and subjective risk perception data from 31 major Chinese cities (2017–2023) within a novel tri-spatial framework, constructing a multidimensional indicator system to quantify PRPB and its spatiotemporal patterns. Furthermore, using multiple linear regression and mediation analysis, we empirically investigate the formation mechanisms of PRPB, incorporating factors such as psychological stress, social capital, media rationality, and government trust. This study makes three key contributions: (1) Theoretically, it breaks new ground by proposing a tri-spatial framework integrating physical, social, and cyber dimensions—advancing beyond existing one- or two-dimensional analytical frameworks and offering novel insights into modern risk cognition. (2) Mechanistically, it overcomes prior overreliance on micro-level factors by establishing a cross-level, multi-path explanatory framework, enabling a more holistic understanding of PRPB formation. (3) Methodologically, it innovatively combines subjective risk perceptions with objective risk indicators to develop a robust PRPB measurement tool, providing valuable support for future research.

The remainder of this paper is structured as follows: Section 2 details the construction of the measurement model, indicator selection, and data sources. Section 3 presents empirical results, including PRPB measurement, mechanism analysis via regression models, and robustness checks. Section 4 summarizes key findings and discusses policy implications. This systematic research design not only advances theoretical and methodological understanding of PRPB but also provides empirical evidence to inform government strategies for developing targeted risk communication systems and enhancing public risk cognition.

## 2 Materials and methods

### 2.1 Extended tri-spatial prism model for public risk perception bias

Social Judgment Theory (SJT) originates from the probabilistic functionalism proposed by Hammond and Adelman (20), which aims to explain the psychological mechanisms underlying individuals’ evaluations and judgments of people, events, and objects in social contexts. This theory is particularly applicable to analyzing the discrepancy between subjective perception and objective reality in policy-making and risk assessment scenarios. Centered on the “lens model” as its core analytical framework, SJT divides the perception system into two key components: (1) the internal “cognitive system” of individuals, which refers to the subjective judgment process based on cognition and experience; and (2) the external “environmental system,” which represents objectively existing realistic conditions and risk attributes. Subsequent studies exemplified by Cooksey (21) incorporated judgment factors such as individual experience and media communication, thereby enhancing the theory’s explanatory power in real-world risk perception contexts.

**Figure 1:**
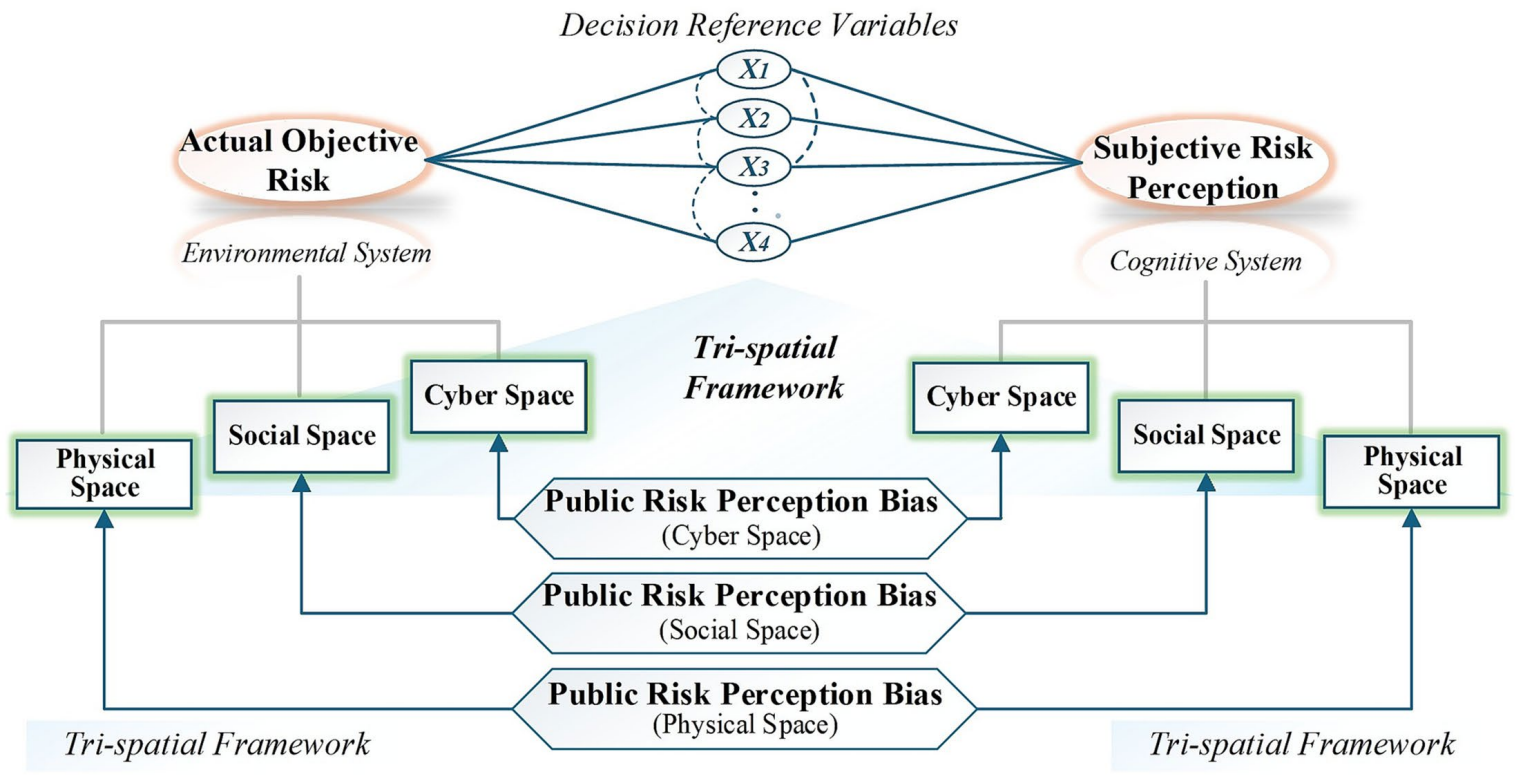
Extended tri-spatial prism model based on tri-spatial framework.

Building on traditional SJT, this study proposes an Extended Tri-Spatial Prism Model grounded in a Tri-Spatial Framework (see Figure 1) to measure PRPB, achieving theoretical advancements in three key aspects: (1) It introduces a novel tri-spatial framework that categorizes PRPB across physical, social, and cyber dimensions, providing a more systematic conceptualization of risk perception; (2) It establishes a quantitative measurement system integrating objective risk indicators with subjective risk perceptions, enabling direct comparison and analysis of their discrepancies; (3) It develops an analytical module to examine the multidimensional pathways influencing PRPB, with a specific focus on mediating mechanisms involving psychological stress, social capital, media rationality, and government trust.

### 2.2 Tri-spatial assessment method for public risk perception bias

#### 2.2.1 Indicator system construction and weighting

Existing research on public safety risk assessment primarily focuses on single spatial dimensions, such as natural disasters (22), social instability (23), ecological degradation (24), community vulnerabilities (25), or cybersecurity threats (26). To address this limitation, this study deconstructs public safety risks based on the tri-spatial framework (physical, social, cyber) and establishes a comprehensive evaluation system to capture their inherent complexity.

Adhering to the principles of systematicity, scientificity, operability, consensus-driven design, and adaptability, we employed the Delphi method via expert consultations to identify core risk types and construct a multi-level indicator system. The Delphi method is a forecasting and decision-making approach that solicits experts’ opinions anonymously. Through multiple rounds of feedback and statistical aggregation, experts’ viewpoints gradually converge to ultimately form a group consensus. Its core function lies in effectively pooling experts’ wisdom through a structured communication process and mitigating subjective biases in group decision-making (27). By assigning weights to indicators, we quantified deviations between subjective risk perceptions and objective risks across the three spaces. The resulting dynamic system supports public safety decision-making by reflecting adaptive risk patterns. The specific steps are as follows:

- Step 1: Delphi consultation (Two rounds)

Eight experts in security risk assessment and urban emergency management participated in two rounds of Delphi consultation. Experts evaluated the importance of secondary indicators using a 5-point Likert scale (1 = extremely unimportant, 2 = unimportant, 3 = neutral, 4 = important, 5 = extremely important). To refine the initial indicator system, secondary indicators with a mean importance score ≥3.5 and a coefficient of variation (CV) ≤ 0.25 were retained. After deletion, correction, and enhancement, the original eight secondary indicators were consolidated into seven.

- Step 2: Expert evaluation of indicator importance

The primary indicators and revised secondary indicators were resubmitted to the same expert panel, which evaluated the relative importance of indicators at the same level using a 1–9 scale. We aggregated the weights assigned by all eight experts and calculated the average weight for each primary and secondary indicator.

- Step 3: Development of tertiary indicators

Secondary indicators were decomposed into an initial pool of tertiary indicators based on expert input. A focus group meeting with the experts was convened to eliminate redundancy, integrate overlapping dimensions, and supplement critical omissions. Logical validity was confirmed by achieving a content validity index (CVI) > 0.80, resulting in a comprehensive set of tertiary indicators covering both subjective risk perceptions and actual objective risks (see Table 1).

- Step 4: Weight determination via entropy weight method (EWM)

**Table 1:** Indicator system for public risk perception in tri-spatial dimensions.

Spatial dimension	Weight	Secondary indicators	Weight	Tertiary indicators (subjective)	Weight	Tertiary indicators (objective)	Weight
Physical space	0.3482	Natural risk	0.4671	Perceived disaster occurrence (+)	0.5153	Frequency of earthquakes (+)	0.0790
						Frequency of typhoons (+)	0.0739
						Frequency of floods (+)	0.3916
				Perceived disaster severity (+)	0.4847	Climate risk index (+)	0.4554
		Ecological risk	0.5329	Pressure on resource shortage (+)	0.4342	Per capita urban park green area (−)	0.0173
						Per capita urban water resources (−)	0.0164
				Perceived environmental degradation (+)	0.5658	Municipal solid waste treatment rate (−)	0.7860
						Annual mean PM₂.₅ concentration (+)	0.0331
						Municipal wastewater treatment rate (−)	0.1273
						Urban green space ratio (−)	0.0199
Social space	0.3875	Public health risk	0.3452	Pressure on infectious diseases (+)	0.5625	Influenza incidence rate (+)	0.2438
						Hepatitis B incidence rate (+)	0.2857
						Infectious disease incidence rate (+)	0.1910
				Pressure on medical resource shortage (+)	0.4375	Number of healthcare institutions per 10,000 people (−)	0.0677
						Number of hospital beds per 1,000 people (−)	0.1116
						Number of physicians per 1,000 people (−)	0.1002
		Public facility risk	0.1347	Pressure on facility failure (+)	0.3993	Length of drainage pipes (−)	0.3191
				Pressure on inadequate facility improvements (+)	0.6007	Annual completed investment in municipal infrastructure (−)	0.6809
		Public security risk	0.2468	Perceived personal safety risk (+)	1.0000	Number of criminal cases (+)	1.0000
		Social Security Risk	0.2733	Pressure on old-age support (+)	0.2810	Pension insurance enrollment per 1,000 people (−)	0.3827
				Pressure on healthcare access (+)	0.3426	Medical insurance enrollment per 1,000 people (−)	0.3613
				Unemployment pressure (+)	0.3311	Unemployment insurance enrollment per 1,000 people (−)	0.3100
Cyber space	0.2643	Cyber security risk	1.0000	Perceived information risk (+)	0.3311	City-level information security index (−)	0.3302
				Pressure on cybercrime occurrence (+)	0.6689	Number of cybercrime cases (+)	0.6698

“+” indicates a positive-oriented indicator (higher values correspond to higher risk); “-” indicates a negative-oriented indicator (higher values correspond to lower risk), which were reverse-coded during data analysis to ensure consistent directionality.

The entropy weight method (EWM), an objective weighting approach, was employed to determine the weights of tertiary indicators. This method assigns weights based on the dispersion of indicator data: a smaller entropy value indicates greater information content and a higher weight, while a larger entropy value corresponds to a lower weight. Concurrently, to mitigate the potential scale effect of the EWM, two measures were adopted. First, following the methodologies of Cheng and Liu (28) and Zhang et al. (29), the Min-Max normalization method was used to linearly transform raw data, which was then uniformly mapped to the [0, 1] interval to eliminate scale discrepancies from differences in indicator dimensions and orders of magnitude. Second, a hierarchical weighting approach was applied to calculate intra-group weights (defined by secondary indicators), thereby reducing the scale effect arising from excessive differences in indicator types. Furthermore, to ensure the temporal comparability of 2017–2023 PRPB data, we followed the common practice of using the entropy weight method for multi-year weight determination (30, 31). Specifically, all annual data were integrated and processed collectively to derive unified weights. The specific steps are as follows:

a) Construct the original data matrix

Assuming there are n samples and m tertiary indicators, the original data matrix is defined Equation 1 as:


$$X=[\begin{array}{ccc} x_{11} & \cdots & x_{1m}\\ \vdots & \cdots & \vdots \\ x_{n1} & \cdots & x_{\mathrm{nm}} \end{array}]$$ (1)

b) Data standardization

Since indicators share consistent dimensions and attributes, uniform dimensionless standardization was applied. Positive indicators (higher values indicate higher risk) and negative indicators (higher values indicate lower risk) were processed using Equations (2, 3), respectively:


$$x\prime_{\mathrm{ij}}=\frac{x_{\mathrm{ij}}-\min(x_{j})}{\max(x_{j})-\min(x_{j})}\;\;\;(\text{Positive indicators})$$ (2)

$$x\prime_{\mathrm{ij}}=\frac{\max(x_{j})-x_{\mathrm{ij}}}{\max(x_{j})-\min(x_{j})}\;\;\;(\text{Negative indicators})$$ (3)

Where $\;x_{\mathrm{ij}}$ is the original value of the *j*-th indicator for the i -th sample, $x\prime_{\mathrm{ij}}$ is the standardized result, and $\min(x_{j})$ and $\max(x_{j})$ are the minimum and maximum values of the *j*-th indicator, respectively. The standardized matrix is:


$$X^{\prime }=[\begin{array}{ccc} x_{11}^{\prime } & \cdots & x_{1m}^{\prime }\\ \vdots & \cdots & \vdots \\ x_{n1}^{\prime } & \cdots & x_{\mathrm{nm}}^{\prime } \end{array}]$$ (4)

- c) Calculate information entropy

The information entropy $e_{j}$ for the j-th indicator is computed as:


$$\begin{array}{c} p_{\mathrm{ij}}=\frac{x\prime_{\mathrm{ij}}}{\sum_{i=1}^{m}x\prime_{\mathrm{ij}}} \end{array}$$ (5)

$$\begin{array}{c} e_{j}=-\frac{\sum_{1}^{m}p_{\mathrm{ij}}\ln(p_{\mathrm{ij}})}{\ln m} \end{array}$$ (6)

$$\begin{array}{c} d_{j}=1-e_{j} \end{array}$$ (7)

Where $p_{\mathrm{ij}}$ is the weight of the *i*-th sample under the *j*-th indicator, and $d_{j}$ is the information entropy redundancy (i.e., the degree of discrimination of the j-th indicator).

d) Calculate indicator weights

The weight $w_{j}$ of the *j*-th tertiary indicator is:


$$\begin{array}{c} w_{j}=\frac{d_{j}}{\sum_{j=1}^{n}d_{j}} \end{array}$$ (8)

Through this process, a tri-spatial PRPB evaluation system was constructed, encompassing both subjective risk perceptions and actual objective risks. The detailed structure is presented in Table 1.

#### 2.2.2 Public risk perception bias measurement model

To quantify PRPB, we first calculated scores for subjective risk perceptions and actual objective risks separately, then computed their discrepancy. The specific models are as follows:

(1) Scoring subjective risk perceptions

a) Score of secondary indicators

The score of the k -th secondary indicator $\left(S_{\mathrm{sub}\_\sec,k}\right)$ is calculated using its weighted tertiary indicators:


$$S_{\mathrm{sub}\_\sec,k}=\sum_{j=1}^{t}x\prime_{\mathrm{sub},j}\cdot w_{\mathrm{sub},j}\;$$ (9)

Where $x\prime_{\mathrm{sub},j}$ is the standardized value of the j -th subjective tertiary indicator under the k -th secondary indicator, $w_{\mathrm{sub},j}$ is the weight of the j -th subjective tertiary indicator, and t is the number of subjective tertiary indicators under the k -th secondary indicator.

b) Score of primary spatial dimensions

The score of the p -th primary spatial dimension ( $S_{\mathrm{sub}\_\mathrm{spa},p}$ ) is calculated using its weighted secondary indicators:


$$S_{\mathrm{sub}\_\mathrm{spa},p}=\sum_{k=1}^{u}S_{\mathrm{sub}\_\sec,k}\cdot w_{\sec,k}$$ (10)

Where $w_{\sec,k}$ is the weight of the k -th secondary indicator under the p -th spatial dimension, and u is the number of secondary indicators under the p -th spatial dimension.

c) Overall subjective risk perception score

The overall subjective risk perception score ( $S_{\mathrm{sub}\_\text{total}}$ ) is calculated using the weighted primary spatial dimensions:


$$S_{\mathrm{sub}\_\text{total}}=\sum_{p=1}^{3}S_{\mathrm{sub}\_\mathrm{spa},p}.w_{\mathrm{spa},p}$$ (11)

Where $w_{\mathrm{spa},p}$ is the weight of the p -th primary spatial dimension (physical, social, cyber; each assigned 0.3333 in this study).

(2) Scoring actual objective risk

The scoring logic for actual objective risks is consistent with that of subjective risk perceptions, with the following equations:

a) Score of secondary indicators

The score of the k -th secondary indicator $\left(S_{\mathrm{obj}\_\sec,k}\right)$ is calculated using its weighted tertiary indicators:


$$S_{\mathrm{obj}\_\sec,k}=\sum_{j=1}^{v}x\prime_{\mathrm{obj},j}\cdot w_{\mathrm{obj},j}\;$$ (12)

Where $x\prime_{\mathrm{obj},j}$ is the standardized value of the j -th objective tertiary indicator under the k -th secondary indicator, $w_{\mathrm{obj},j}\;$ is the weight of the j -th objective tertiary indicator, and v is the number of subjective tertiary indicators under the *k* -th secondary indicator.

b) Score of primary spatial dimensions

The score of the p -th primary spatial dimension ( $S_{\mathrm{obj}\_\mathrm{spa},p})$ is calculated using its weighted secondary indicators:


$$S_{\mathrm{obj}\_\mathrm{spa},p}=\sum_{k=1}^{u}S_{\mathrm{obj}\_\sec,k}\cdot w_{\sec,k}$$ (13)

Where $w_{\sec,k}$ is the weight of the k -th secondary indicator under the p -th spatial dimension, and u is the number of secondary indicators under the p -th spatial dimension.

c) Overall actual objective risk score

The overall actual objective risk score ( $S_{\mathrm{obj}\_\text{total}}$ ) is calculated using the weighted primary spatial dimensions:


$$S_{\mathrm{obj}\_\text{total}}=\sum_{p=1}^{3}S_{\mathrm{obj}\_\mathrm{spa},p}.w_{\mathrm{spa},p}$$ (14)

Where $w_{\mathrm{spa},p}$ is the weight of the p -th primary spatial dimension (i.e., physical space, social space, and cyber space).

(3) Calculation of public risk perception bias

Drawing on Martin (32), a PRPB calculation model was constructed using the difference method. Specifically, PRPB is defined as the discrepancy between the overall actual objective risk score ( $S_{\mathrm{obj}\_\text{total}}$ ) and the overall subjective risk perception score ( $S_{\mathrm{sub}\_\text{total}}$ ):


$$\text{Bias}=S_{\mathrm{obj}\_\text{total}}-S_{\mathrm{sub}\_\text{total}}$$ (15)

Where Bias is the risk perception bias value. If Bias > 0, the public’s risk perception exhibits an optimism bias (risk underestimation bias). Conversely, if Bias < 0, the public’s risk perception exhibits a pessimism bias (risk overestimation bias).

### 2.3 Multi-level mechanisms of public risk perception bias

**Figure 2:**
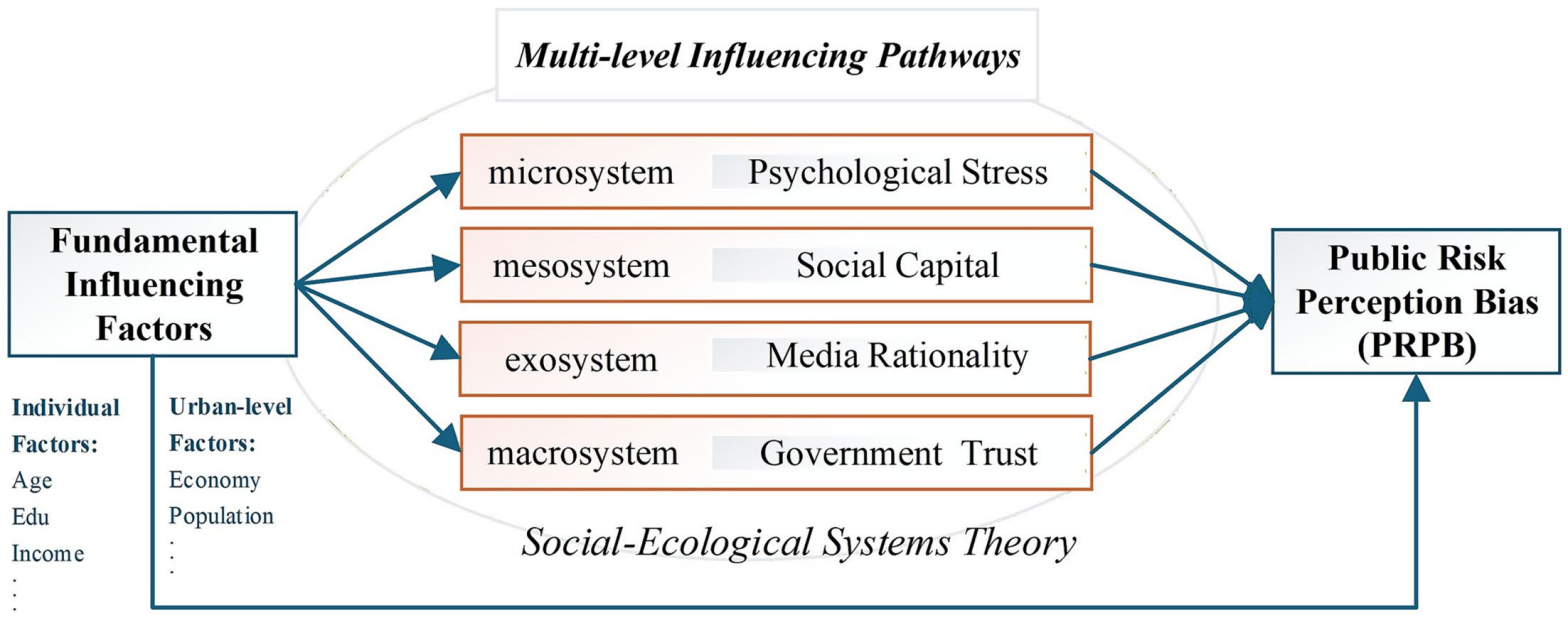
Multi-level influencing mechanisms of public risk perception bias.

Existing research indicates that individual factors such as education level (33), age (34), and income (35) significantly affect PRPB, and urban economic and social development influences PRPB by altering exposure probability and resource availability for response (36). Additionally, grounded in Social-Ecological Systems Theory (37), this study develops a multi-level pathway model to unpack the influencing mechanisms of PRPB within the tri-spatial framework (physical, social, and cyber spaces). The model operates across four nested levels—microsystem, mesosystem, exosystem, and macrosystem—while excluding the chronosystem (temporal dynamics) to maintain focus on cross-sectional socio-psychological pathways (see Figure 2). The theoretical logic of each level is as follows:

At the microsystem level, guided by Protection Motivation Theory (PMT), excessive emergency emotions (e.g., panic) at the individual level elevate threat appraisal but reduce coping appraisal. This psychological imbalance leads individuals to overestimate risk severity and personal susceptibility, while underestimating self-efficacy and response effectiveness (38)—ultimately manifesting as a pessimism bias.

At the mesosystem level, social capital (e.g., social networks, collective trust) fosters optimism bias by enhancing collective risk response efficacy. It achieves this through two key pathways: filtering misinformation via strong social ties (e.g., family, close communities) and dispersing risk uncertainties via weak ties (e.g., community organizations, neighborhood networks) (39).

At the exosystem level, media rationality (i.e., the public’s ability to discern credible information from media sources) cultivates moderate optimism by strengthening information discernment, reducing susceptibility to rumors, and mitigating the “availability heuristic” (40).

At the macrosystem level, government trust enhances optimistic risk expectations through two institutional mechanisms: credible policy commitments (e.g., clear risk mitigation policies) and transparent emergency response processes (e.g., timely risk information disclosure) (41).

This integrative framework reveals that PRPB is not driven by isolated factors but by synergistic interactions among individual psychological traits, regional social contexts, and multi-level factors in social-ecological systems.

To empirically validate the proposed multi-level influencing mechanisms, this study employs the three-step mediation analysis approach developed by Baron and Kenny (42). Specifically, core explanatory variables including gender (43), age (34), and education level (33) are commonly treated as predetermined or exogenous variables, while psychological stress, social capital, media rationality, and government trust are designated as mediating variables. This classification is rooted in the observation that the latter set of variables may be shaped by demographic characteristics and urban social development levels—for example, urban population density may influence social capital, and residents’ education levels may also affect government trust. In turn, these mediating variables serve as a transmission mechanism linking core explanatory variables to the dependent variable (PRPB), with the detailed steps and equations presented as follows:

First, we test the total effect of fundamental influencing factors ( X ) on PRPB ( Y ). The regression equation is:


$$Y=\beta_{0}+\beta_{1}X+\varepsilon_{1}$$ (16)

Where $\beta_{1}$ represents the total effect of X on Y , $\beta_{0}$ is the intercept, and $\varepsilon_{1}$ is the random error term. A significant $\beta_{1}$ confirms that X has a direct association with Y , which is a prerequisite for subsequent mediation tests.

Next, we test the effects of the fundamental influencing factors (X) on each mediator ( M , including psychological stress, social capital, media rationality, and government trust). As presented in Equation 17, the regression model is:


$$M=\alpha_{0}+\alpha_{1}X+\varepsilon_{2}$$ (17)

Where $\alpha_{1}$ denotes the effect of X on M , $\alpha_{0}$ is the intercept, and $\varepsilon_{2}$ is the random error term. A significant $\alpha_{1}$ indicates that X can significantly predict changes in M , satisfying the second condition for mediation.

Finally, we estimate the joint impact of X and M on Y by including both variables in the regression model. The Equation 18 is:


$$Y=\gamma_{0}+\gamma_{1}X+\gamma_{2}M+\varepsilon_{3}$$ (18)

Where $\gamma_{1}$ represents the direct effect of X on Y after controlling for M , $\gamma_{2}$ denotes the net effect of M on Y , $\gamma_{0}$ is the intercept, and $\varepsilon_{3}$ is the random error term.

Mediation is confirmed if: (1) $\alpha_{1}$ and $\gamma_{2}$ are both significant; and (2) $\gamma_{1}$ is smaller in magnitude than $\beta_{1}$ (partial mediation) or becomes non-significant (full mediation). To further validate the robustness of mediation effects, we also conduct bootstrapping tests (with 5,000 resamples) to calculate 95% confidence intervals for the indirect effect ( $\alpha_{1}\times \gamma_{2}$ )—a non-zero confidence interval confirms the statistical significance of the mediation pathway.

### 2.4 Variable measurement and data sources

#### 2.4.1 Measurement of subjective risk perception

Subjective risk perception data were derived from the “Annual Survey of Urban Public Safety Perception in China” (2017–2023), an annual tracking project conducted by the Institute of Emergency Management and National Security at China University of Mining and Technology. The survey adopted a nationwide sampling design covering 31 provincial capitals and major cities, with respondents rating their risk perceptions using a 10-point Likert scale (1 = “not concerned at all” to 10 = “extremely concerned”). After rigorous data cleaning (e.g., removing missing values, outliers, and inconsistent responses), 55,665 valid samples were retained, forming a nationally representative dataset. Specific indicators for each spatial dimension are detailed below:

(1) Physical space subjective risk perception

Natural risk: Measured by two items: (i) “To what extent are you concerned that natural disasters (e.g., earthquakes, floods, droughts, typhoons) in your city may cause life or property losses?” (Perceived disaster occurrence, +); (ii) “To what extent are you worried that natural disaster issues in your city are worsening?” (Perceived disaster severity, +).

Ecological risk: Measured by two items: (i) “To what extent do you worry that resource shortages and ecological imbalances (e.g., water scarcity, wetland reduction, biological chain disruption) threaten urban development?” (Pressure on resource shortage, +); (ii) “To what extent are you concerned that your city’s ecological environment is deteriorating?” (Perceived environmental degradation, +).

(2) Social space subjective risk perception

Public health risk: Measured by two items: (i) “To what extent are you worried about potential outbreaks of infectious diseases (e.g., AIDS, tuberculosis, COVID-19) around you?” (Pressure on infectious diseases, +); (ii) “To what extent do you worry about not receiving timely and effective treatment during an infectious disease outbreak?” (Pressure on medical resource shortage, +).

Public facility risk: Measured by two items: (i) “To what extent do you worry about failures of municipal public facilities (e.g., manhole covers, sewage systems, elevators, gas pipelines)?” (Pressure on facility failure, +); (ii) “To what extent are you concerned that safety improvements for public spaces will not be implemented?” (Pressure on inadequate facility improvements, +).

Public security risk: Measured by two items: (i) “To what extent do you feel anxious about personal safety when traveling alone at night?” (Perceived personal safety risk, +); (ii) “To what extent do you fear being harmed by nearby violent conflicts?” (Perceived personal safety risk, +).

Social security risk: Measured by three items: (i) “To what extent do you worry about financial support and care in old age?” (Pressure on old-age support, +); (ii) “To what extent are you concerned about affording medical treatment when ill?” (Pressure on healthcare access, +); (iii) “To what extent do you worry that families facing unexpected difficulties (e.g., unemployment, illness, accidents) will not receive necessary assistance?” (Unemployment pressure, +).

(3) Cyber space subjective risk perception

Cyber security risk: Measured by three items: (i) “To what extent are you concerned about not receiving effective help when encountering information harassment or fraud?” (Perceived information risk, +); (ii) “To what extent do you fear that personal privacy (e.g., phone number, ID, address) will be stolen for commercial or criminal purposes?” (Perceived information risk, +); (iii) To what extent are you worried about the increasing prevalence of information crimes (e.g., hacking, virus attacks, financial fraud)?” (Pressure on cybercrime occurrence, +).

#### 2.4.2 Measurement of actual objective risks

Actual objective risk indicators were selected based on their alignment with subjective risk dimensions, with data sourced from official statistical databases and authoritative research institutions to ensure reliability. Specific indicators are as follows:

(1) Physical space objective risk

Natural risk: (i) Frequency of earthquakes/typhoons/floods (+): Annual count of earthquakes (≥4.0 magnitude), typhoons (≥Level 8), and floods (recorded by local flood control departments), sourced from the Earth Resources Data Cloud; (ii) Climate Risk Index (+): Composite index reflecting regional climate vulnerability (e.g., extreme temperature, precipitation anomalies), sourced from the China Meteorological Administration.

Ecological risk: (i) Per capita urban park green area (−), Per capita urban water resources (−) and Urban green space ratio (−) sourced from the *China Urban Construction Statistics Yearbook*; (ii) Annual mean PM2.5 concentration (+), Municipal solid waste treatment rate (−), and Municipal wastewater treatment rate (−) sourced from the *China Statistical Yearbook*.

(2) Social space objective risk

Public health risk: (i) Influenza/ Hepatitis B/ Infectious disease incidence rate (+): Given the scarcity of city-level infectious disease incidence data, this study adopts the method of Huang et al. (44), Fang et al. (45), and Wang et al. (46), using the search volume index (sourced from Baidu Index) that reflects population-level search behavior related to infectious diseases as an objective proxy for the actual incidence of influenza, Hepatitis B, and other infectious diseases. Specifically, the data were min-max standardized, converting absolute search volumes into relative rankings within respective series to mitigate measurement volatility and potential proxy variable-induced distortion. (ii) Number of hospitals/health centers, hospital beds, and doctors per 1,000 people (−) sourced from the *China Statistical Yearbook*.

Public facility risk: (i) Length of drainage pipes per 100 km^2^ (−); (ii) Annual completed investment in municipal infrastructure (100 million yuan) (−). Both are sourced from the *China Urban Construction Statistics Yearbook*.

Public security risk: Number of criminal cases per 1,000 people (+) sourced from annual reports of municipal courts and procuratorates. Meanwhile, min-max standardization was employed to render crime rates comparable by relative positioning, which helps alleviate the risk underestimation resulting from potential underreporting.

Social security risk: Pension/Medical/Unemployment insurance enrollment per 1,000 people (−) sourced from the *China Statistical Yearbook*.

(3) Cyber space objective risk

Cybersecurity risk: (i) City-level information security index (−): Composite index evaluating urban information security governance (e.g., cybersecurity infrastructure, incident response capability), sourced from the Institute of Digital China Research; (ii) Number of cybercrime cases per 1,000 people (+) sourced from annual reports of municipal judicial institutions. The data on cybercrime cases were also standardized via the min-max method to mitigate the potential underestimation of objective risks.

#### 2.4.3 Measurement of influencing factors for public risk perception bias

Building on Winkleby et al. (35) and Ando et al. (16), we incorporate individual and urban-level variables as fundamental influencing factors. Additionally, mediating variables are included based on social-ecological systems theory, with their measurements and data sources detailed as follows:

(1) Individual factors

Measured using categorical or ordinal scales, with data sourced from the 2023 wave of the “Annual Survey of Urban Public Safety Perception in China.”

Gender: 0 = “Male,” 1 = “Female.”

Age: 1 = “18–29 years,” 2 = “30–44 years,” 3 = “45–59 years,” 4 = “≥60 years.”

Hukou Type (household registration): 1 = “Rural migrant hukou,” 2 = “Urban migrant hukou,” 3 = “Local rural hukou,” 4 = “Local urban hukou.”

Education Level: 1 = “Primary school or below,” 2 = “Junior high school,” 3 = “Senior high school/vocational school,” 4 = “bachelor’s degree,” 5 = “Postgraduate or above.”

Income Level (monthly household income per capita): 1 = “<¥2,000,” 2 = “¥2,001–3,500,” 3 = “¥3,501–5,000,” 4 = “¥5,001–8,000,” 5 = “¥8,001–12,000,” 6 = “>¥12,000.”

(2) Urban-level factors

*Per Capita* GDP (10,000 yuan): Reflecting urban economic development level, sourced from the *China Statistical Yearbook*.

Population Density (people per km^2^): Reflecting urban social development conditions, sourced from the *China Statistical Yearbook*.

(3) Mediating variables

All mediating variables were measured using a 10-point Likert scale (1 = “strongly disagree” to 10 = “strongly agree”) with data from the 2023 “Annual Survey of Urban Public Safety Perception in China.” Confirmatory Factor Analysis (CFA) was conducted to verify construct validity, with all Cronbach’s *α* > 0.7, indicating good internal consistency:

Psychological Stress (microsystem): (i) “When I learn about serious public safety incidents, I often feel angry”; (ii) “When I learn about serious public safety incidents, I often feel afraid.”

Social Capital (mesosystem): (i) “I believe interpersonal relationships in the current social environment are good”; (ii) “I think the community where I live is friendly.”

Media Rationality (exosystem): (i) “I pay high attention to hot public safety events”; (ii) “I can independently assess the reliability of public safety information.”

Government Trust (macrosystem): (i) “I trust that government agencies proactively take measures to protect residents from public safety threats”; (ii) “I believe the government has sufficient capability to address urban public safety issues”; (iii) “I trust that government departments make every effort to respond to public safety threats.”

## 3 Results

### 3.1 Assessment results of public risk perception bias

#### 3.1.1 Measurement of public risk perception bias: pessimism vs. optimism

Based on the measurement of PRPB across the tri-spatial dimensions (2017–2023), the overall sample exhibited a mean bias value of −0.132 (SD = 0.142), indicating a systematic pessimism bias—i.e., the public systematically overestimates actual risks (see Table 2). The distribution of this bias showed significant asymmetry: pessimism bias dominated (87% of observations, *n* = 188), with deviations ranging from mild to severe (min = −0.559), while optimism bias was less prevalent (13%, *n* = 29; max = 0.283).

**Table 2:** Measurement results of public risk perception bias (2017–2023).

Public risk perception bias	Observations	Mean	Standard deviation	Max	Min	Optimism bias (*n*, %)	Pessimism bias (*n*, %)
Overall Level	217	−0.132	0.142	0.283	−0.559	29 (13%)	188 (87%)
Physical Space	217	−0.231	0.224	0.466	−0.814	35 (16%)	182 (84%)
Social Space	217	0.015	0.197	0.467	−0.619	109 (50%)	108 (50%)
Cyber Space	217	−0.215	0.203	0.549	−0.828	26 (12%)	191 (88%)

This pattern can be attributed to several socio-psychological mechanisms aligned with prior theoretical frameworks: (1) Media-driven “availability heuristics,” where frequent coverage of negative risk events amplifies the recall of adverse outcomes (47); (2) Negative information preference in social learning, a cognitive tendency to prioritize threatening information over neutral or positive content (48); (3) Perceived lack of control in complex urban systems, where individuals adopt risk pessimism as a psychological coping strategy (49). Notably, the minority optimism bias may stem from the buffering effect of government trust, but its potential to induce risk optimism warrants cautious attention in risk governance.

**Figure 3:**
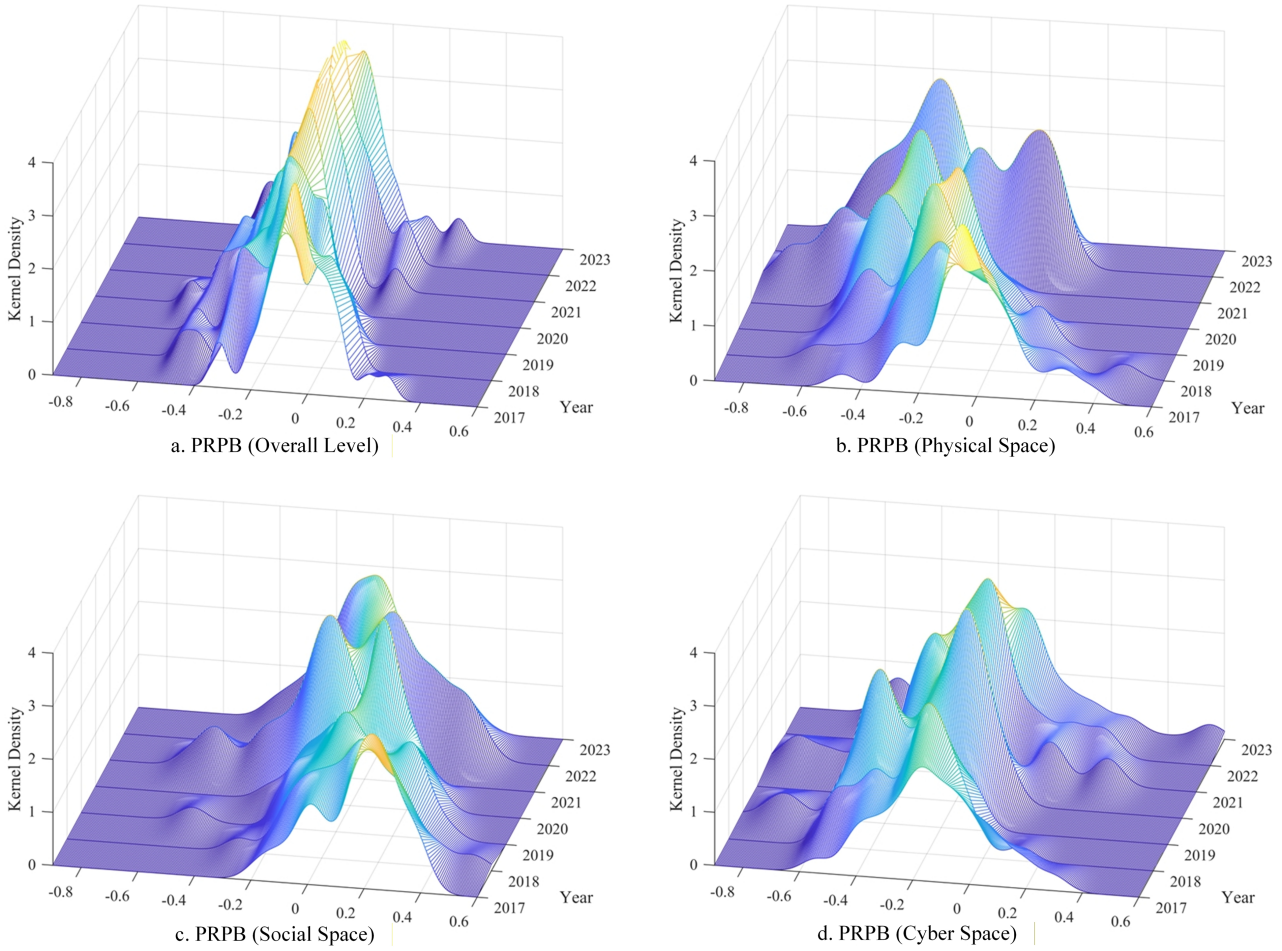
Kernel density of public risk perception bias (2017–2023).

To further explore the distribution characteristics of PRPB, kernel density plots were generated (see Figure 3). Consistent with the tri-spatial framework, PRPB exhibits significant spatial heterogeneity, with detailed analyses presented below:

Further analysis of the overall level kernel density plot for PRPB (see Figure 3) reveals that the kernel density curve shifted leftward from 2017 to 2021 and rightward from 2021 to 2023, indicating that PRPB exhibited a sustained pessimism trend during 2017–2021, followed by a gradual shift toward optimism post-2021. Meanwhile, the peak intensity of the curve showed an overall upward trend over the observation period, evolving progressively from an initial “multi-peak” to a “single-peak” distribution. This reflects continuous improvement in PRPB concentration, indicating a steady increase in public consensus on risk perception.

In physical space, a strong systematic pessimism bias prevailed (*M* = −0.231, SD = 0.224), with 84% of observations showing pessimism (see Table 2). This may align with the mediating role of media in risk amplification (18), as frequent natural disasters (e.g., floods, typhoons) and their extensive media coverage reinforce negative risk perceptions. Further analysis of the kernel density curve in physical space (see Figure 3) reveals a distinct “multi-peak” structure, indicating a low concentration of PRPB. This is primarily attributed to two factors: first, the uneven spatial distribution and varying intensities of natural disasters result in heterogeneous risk exposure among residents across regions; second, significant disparities exist among local communities in risk prevention capacity and public risk communication quality, further impeding the formation of a concentrated distribution pattern of PRPB.

In social space, PRPB maintained an equilibrium state, with a slight optimism bias (*M* = 0.015, SD = 0.197) and an equal 50% split between optimistic and pessimistic observations (see Table 2). The kernel density curve for PRPB in social space was slightly right-skewed, with stable peak density across years (see Figure 3), a characteristic that further corroborates the above finding. This equilibrium may stem from social space’s unique attributes: (1) The lagged manifestation of social risks (e.g., pension gaps) mitigates immediate emotional responses (50); (2) Diversified information channels (e.g., government bulletins, community discussions) correct extreme biases (51); (3) “Collective wisdom” in social groups offsets individual biases through varied risk-decoding abilities (52). This balance differs from the systematic biases in physical/cyber spaces, reflecting the complexity of social spatial risk cognition.

In cyber space, an even more pronounced pessimism bias was observed (*M* = −0.215, SD = 0.203), with 88% of observations showing pessimism (see Table 2). Further analysis of the cyber space PRPB kernel density plot reveals a wide distribution of values, indicating fragmented collective perception. This pronounced pessimism bias may be driven by growing public concerns over data security breaches, online fraud, and the inflation of hypothetical cyber risks (e.g., algorithmic discrimination) in public discourse (53). Meanwhile, such fragmented collective perception underscores the imperative of targeted risk communication to mitigate extreme biases and foster more coherent risk cognition.

#### 3.1.2 Spatiotemporal patterns of public risk perception bias

(1) Temporal evolution of public risk perception bias

**Figure 4:**
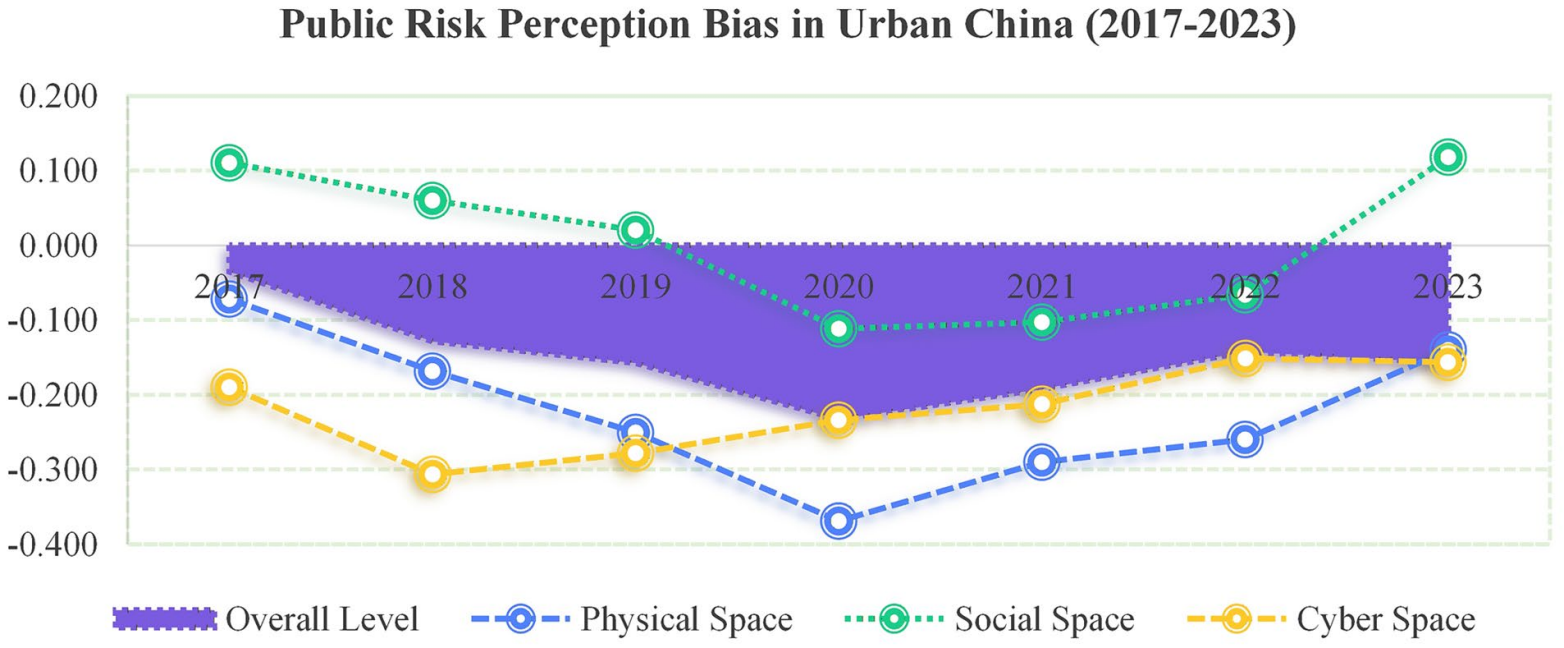
Temporal evolution of public risk perception bias (2017–2023).

Across the tri-spatial framework, PRPB followed a distinct “V-shaped” temporal trajectory from 2017 to 2023 (see Figure 4), with phase-specific drivers aligned with external shocks and policy interventions.

In physical space, PRPB deteriorated from −0.069 (2017) to −0.364 (2020), which may be driven by ecological pollution and frequent natural disasters during rapid urbanization, as well as social media’s amplification of pessimistic cognition (54). Post-2020, policy interventions (e.g., optimized ecological governance, improved disaster response systems) facilitated a recovery to −0.152 (2023), demonstrating the effectiveness of institutional measures in mitigating pessimism.

The fluctuations of PRPB in social spaces were closely tied to the COVID-19 pandemic: (1) 2017–2019: A stable optimism bias prevailed (*β* = 0.025); (2) 2020: A sharp shift to pessimism (*β* = −0.061) occurred, driven by public anxiety and eroded government trust due to exposed public health system vulnerabilities during the COVID-19 pandemic (55, 56). (3) 2021–2023: A rebound to optimism (*β* = 0.087) was observed, validating the “shock-adaptation-recovery” trajectory of resilience theory (57)—enhanced risk communication and social capital rebuilding restored public confidence.

PRPB in cyber space has evolved through two distinct phases: (1) Pre-2018: Pessimism (*M* = −0.302 in 2018) heightened due to online fraud, privacy breaches, and regulatory gaps; (2) Post-2018: Regulatory initiatives—including the EU’s GDPR (58), China’s “Clean Internet Action,” and the Cybersecurity Level Protection Standard (GB/T 22239-2019) (59)—drove an average annual improvement of 27.6% in PRPB. However, emerging threats (e.g., algorithmic bias, deepfake fraud) maintained a mild pessimism bias (−0.156 in 2023), highlighting ongoing challenges in cyber risk governance.

(2) Spatial distribution of public risk perception bias

**Figure 5:**
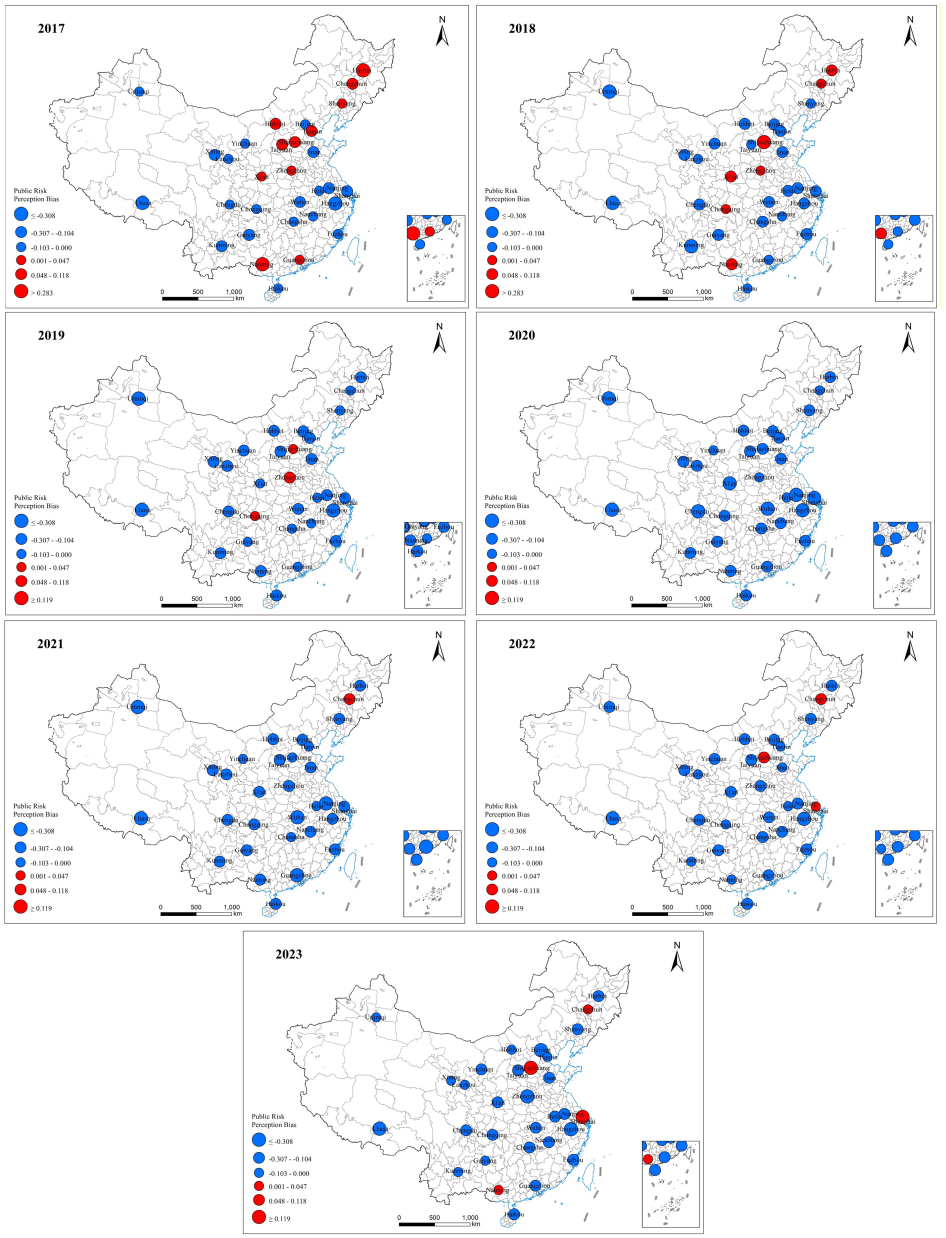
Spatial distribution of public risk perception bias (2017–2023).

Spatial patterns of PRPB exhibited dynamic regional reconstruction during the study period (see Figure 5).

In 2017, a “North-optimistic, South-pessimistic” divergence in PRPB emerged across China. Northeastern and Northern regions (e.g., Harbin: 0.283; Shijiazhuang: 0.116; Taiyuan: 0.118) exhibited significant optimism, while Southern China (e.g., Hangzhou: −0.308; Nanjing: −0.139) displayed pessimism. This spatial pattern may stem from differentiated interactions between regional development, government trust, social capital, and population mobility. Northern cities (e.g., Harbin, Shijiazhuang) exhibit systemic optimism, potentially underpinned by the mutual reinforcement of stable social capital and government trust. The government’s central governance role fosters high public trust (60, 61), while low population mobility facilitates the formation of strong-tie social capital (62), jointly buffering collective risks. In contrast, pessimism bias in developed Southern cities (e.g., Hangzhou, Nanjing) may arise from dual compounding effects: intense urbanization increases risk exposure, and high population density likely exacerbates resource and infrastructure pressures (63). Concurrently, the expansion of highly educated cohorts may shift public focus toward critical scrutiny of “slow-variable risks” (e.g., ecological degradation, long-term security) (64). The interaction between this forward-looking cognition and urban vulnerability constitutes a plausible mechanism driving pessimism bias.

The COVID-19 pandemic in 2020 triggered a notable nationwide shift toward pessimism in PRPB, significantly altering existing regional patterns. Northern cities like Harbin saw a sharp decline in PRPB from 0.283 to −0.165, while southern cities such as Nanjing dropped further from −0.139 to −0.462. This growing pessimistic trend underscores the profound psychological impact of major public health emergencies on public risk perception, transcending previously entrenched socio-economic and regional differences.

By 2023, the post-pandemic recovery for PRPB varied by region, reflecting differences in safety governance efficacy and urban resilience. Northern cities like Shijiazhuang (0.232) and Changchun (0.009) exhibited a clear rebound in optimism bias, a shift attributable to community-level governance innovations. In Changchun, a multi-level linkage mechanism integrating grids, units, and building floors enhanced community governance resilience, while Shijiazhuang improved public service efficiency and subsequently public trust through integrated social work and volunteer collaboration. Southern cities like Shanghai have reversed pessimism (0.119), demonstrating strong urban resilience through an advanced risk management system. Nanning displayed initial optimism (0.009), indicating a nascent recovery. In contrast, Zhengzhou has sharply declined from mild optimism (0.047 in 2017) to severe pessimism (−0.429 in 2023), revealing systemic vulnerabilities exposed by the “7·20” flood in 2021 and long-term erosion of public trust. These post-2020 trends illustrate how economic resilience, governance quality, and social culture shape public risk perception.

### 3.2 Results of the influencing mechanism test

#### 3.2.1 Analysis of fundamental influencing factors on public risk perception bias

Table 3 presents the benchmark regression results based on Equations 2–16, examining the associations between individual and urban-level factors with PRPB. At the overall level, per capita GDP (*β* = 0.072, *p* < 0.001), residents’ age (*β* = 0.010, *p* < 0.001), and local household registration status (*β* = 0.011, *p* < 0.001) are positively correlated with PRPB. In contrast, urban population density (*β* = −0.007, *p* < 0.01), residents’ education level (*β* = −0.012, *p* < 0.001), and female gender (*β* = −0.051, *p* < 0.001) significantly show a negative correlation with PRPB.

**Table 3:** Results of benchmark regression analysis.

Public risk perception bias	(1) Overall level	(2) Physical space	(3) Social space	(4) Cyber space
*Per Capita* GDP	0.072^***^	0.064^***^	0.078^***^	0.075^***^
	(0.008)	(0.010)	(0.009)	(0.011)
Population density	−0.007^**^	0.003	−0.009^***^	−0.017^***^
	(0.003)	(0.003)	(0.003)	(0.004)
Gender	−0.051^***^	−0.038^***^	−0.065^***^	−0.046^***^
	(0.005)	(0.005)	(0.005)	(0.006)
Age	0.010^***^	0.006^*^	0.017^***^	0.007^*^
	(0.003)	(0.003)	(0.003)	(0.004)
Hukou type	0.011^***^	0.011^***^	0.014^***^	0.007^**^
	(0.002)	(0.002)	(0.002)	(0.003)
Education level	−0.012^***^	−0.008^**^	−0.004	−0.029^***^
	(0.003)	(0.004)	(0.003)	(0.004)
Income level	0.002	0.002	0.004^**^	−0.001
	(0.002)	(0.002)	(0.002)	(0.002)
Constant	−0.795^***^	−0.782^***^	−0.708^***^	−0.941^***^
	(0.089)	(0.106)	(0.095)	(0.118)
*N*	9,146	9,146	9,146	9,146

^***^, ^**^, and ^*^ represent significance levels of 1, 5, and 10%, respectively.

A tri-spatial analysis reveals varied effects of factors across physical, social, and cyber spaces: population density is positively correlated with PRPB in social space (*β* = −0.009, *p* < 0.01) and cyber space (*β* = −0.017, *p* < 0.01), with no significant correlation in physical space (*β* = 0.003, *p* > 0.10). Education level is positively correlated with PRPB in physical space (*β* = −0.008, *p* < 0.05) and cyber space (*β* = −0.029, *p* < 0.01) but shows no significant correlation in social space (*β* = −0.004, *p* > 0.10). Conversely, resident Income level uniquely shows a positive correlation with PRPB in social spaces (*β* = 0.004, *p* < 0.05).

These patterns reflect the complex socio-psychological influencing mechanisms of PRPB, aligning with established theories. On one hand, optimism bias arises from several factors: residents in more developed cities perceive risks as more controllable due to enhanced safety infrastructure and emergency resources; older individuals tend to make more accurate risk assessments based on their extensive risk experience (65); and residents often possess a stronger sense of community belonging compared to outsiders, which fosters optimism bias through a reliable support network. On the other hand, pessimism bias arises through distinct pathways: high population density in urban areas heightens perceived risks due to resource competition and crowding; well-educated individuals demonstrate heightened concern regarding risks due to systematic analysis of long-term threats; women are inclined to report higher anxiety owing to increased sensitivity toward potential dangers (66); and high-income groups tend to maintain an optimism bias in social space due to their economic advantages and access to information.

#### 3.2.2 Analysis of mediating pathways for public risk perception bias

Mediation tests (see Table 4) confirm that PRPB is significantly correlated with four multi-level socio-psychological pathways: psychological stress (microsystem), social capital (mesosystem), media rationality (exosystem), and government trust (macrosystem). Specifically, psychological stress is negatively correlated with PRPB (*β* = −0.028, *p* < 0.001), while social capital (*β* = 0.125, *p* < 0.001), media rationality (*β* = 0.047, *p* < 0.001), and government trust (*β* = 0.136, *p* < 0.001) are all positively correlated with PRPB. Detailed mechanisms for each mediator are as follows:

(1) Psychological stress (microsystem mediator)

**Table 4:** Results of Mediating Pathways Analysis.

Variable	(1) Psychological stress	(2) PRPB	(3) Social capital	(4) PRPB	(5) Media rationality	(6) PRPB	(7) Government trust	(8) PRPB
Mediator variable		−0.028^***^		0.125^***^		0.047^***^		0.136^***^
		(0.011)		(0.010)		(0.011)		(0.009)
*Per Capita* GDP	−0.063	0.072^***^	0.010	0.071^***^	0.014	0.072^***^	0.021^**^	0.070^***^
	(0.091)	(0.008)	(0.009)	(0.008)	(0.008)	(0.008)	(0.009)	(0.008)
Population density	−0.019	−0.007^**^	0.009^***^	−0.008^***^	0.006^**^	−0.007^**^	0.004	−0.007^**^
	(0.014)	(0.003)	(0.003)	(0.003)	(0.003)	(0.003)	(0.003)	(0.003)
Gender	0.016^***^	−0.051^***^	0.005	−0.051^***^	0.003	−0.051^***^	0.002	−0.051^***^
	(0.004)	(0.005)	(0.005)	(0.005)	(0.005)	(0.005)	(0.005)	(0.004)
Age	0.001	0.010^***^	0.003	0.010^***^	−0.002	0.010^***^	0.004	0.010^***^
	(0.003)	(0.003)	(0.003)	(0.003)	(0.003)	(0.003)	(0.003)	(0.003)
Hukou type	−0.006^***^	0.011^***^	0.010^***^	0.010^***^	0.009^***^	0.011^***^	0.002	0.011^***^
	(0.002)	(0.002)	(0.002)	(0.002)	(0.002)	(0.002)	(0.002)	(0.002)
Education level	−0.000	−0.012^***^	−0.008^**^	−0.011^***^	0.016^***^	−0.013^***^	−0.006^*^	−0.011^***^
	(0.003)	(0.003)	(0.003)	(0.003)	(0.003)	(0.003)	(0.003)	(0.003)
Income level	0.002	0.002	−0.001	0.002	0.002	0.002	−0.000	0.002
	(0.002)	(0.002)	(0.002)	(0.002)	(0.002)	(0.002)	(0.002)	(0.002)
Constant	0.525^**^	−0.812^***^	0.539^***^	−0.863^***^	0.458^***^	−0.817^***^	0.434^***^	−0.854^***^
	(0.211)	(0.089)	(0.095)	(0.088)	(0.088)	(0.089)	(0.100)	(0.088)
*N*	9,146	9,146	9,146	9,146	9,146	9,146	9,146	9,146

^***^, ^**^, and ^*^ represent significance levels of 1, 5, and 10%, respectively.

Psychological stress functions as a critical microsystem mediator of risk perception, with gender (female: *β* = 0.016, *p* < 0.001) significantly showing a positive correlation with psychological stress, consistent with neuroendocrine mechanisms where women exhibit stronger stress responses to sudden threats (67). Conversely, local household registration shows a negative correlation with psychological stress (*β* = −0.006, *p* < 0.001), reflecting the stabilizing role of social integration and residency security in mitigating stress (68). Critically, psychological stress significantly shows a negative correlation with PRPB (*β* = −0.028, *p* < 0.001), supporting the “emotion-cognition” integration model of risk perception, where negative emotions distort cognitive assessments of risk (69).

(2) Social capital (mesosystem mediator)

Social capital, operating as a mesosystem mediator, is significantly related to two key factors: population density (*β* = 0.009, *p* < 0.001), which facilitates stronger network ties through frequent interaction, consistent with Social Interaction Theory (70); and local household registration (*β* = 0.010, *p* < 0.01), which reinforces shared identity and participatory norms as predicted by Social Identity Theory (71). Conversely, education level shows a negative correlation with social capital (*β* = −0.008, *p* < 0.01), supporting Putnam’s (72) “substitution effect” wherein human capital acquisition displaces investment in relational networks. Crucially, social capital shows a positive correlation with PRPB (*β* = 0.125, *p* < 0.001), as robust support systems buffer perceived threats and promote positive cognitive appraisal of risks (73).

(3) Media rationality (exosystem mediator)

Media rationality is positively related to three key predictors: population density (*β* = 0.006, *p* < 0.01), which supports rational information processing through advanced urban information infrastructure such as high-speed internet; local household registration (*β* = 0.009, *p* < 0.001), which facilitates access to trusted and community-based information channels; and education level (*β* = 0.016, *p* < 0.001), which enhances critical thinking capacity for evaluating media content. Crucially, media rationality significantly shows a positive correlation with PRPB (*β* = 0.047, *p* < 0.001), as it reduces reliance on emotional heuristics and sensationalized narratives, thereby fostering more calibrated and rational risk assessments.

(4) Government trust (macrosystem mediator)

Government trust is significantly enhanced by two factors: population density (*β* = 0.006, *p* < 0.01), as densely populated economic centers typically possess enhanced public service capabilities (e.g., efficient emergency response), thereby enhancing institutional confidence in the government; and local household registration (*β* = 0.009, *p* < 0.001), reflecting more frequent and positive interactions between residents and government agencies. In contrast, education level is negatively correlated with government trust (*β* = −0.006, *p* < 0.05), aligning with Mauk’s (74) theory that educated groups tend to conduct more critical assessments of governance effectiveness and policy trade-offs. Significantly, higher levels of government trust are associated with a significant increase in the optimism bias regarding PRPB (*β* = 0.136, *p* < 0.001). This finding corroborates Li et al.’s (75) research, indicating that confidence in institutional risk management capabilities fosters a systematic positive bias in the public’s perception of safety.

### 3.3 Results of robustness tests

#### 3.3.1 Robustness test for benchmark regression

To verify the reliability of the baseline regression results, this study employed two complementary robustness checks to address potential biases from outliers and distributional sensitivity: (1) a 2% winsorization of the PRPB variable, truncating extreme values at the 2nd and 98th percentiles to mitigate the influence of outliers (76); and (2) quantile regression estimated at the median, which captures the central tendency of the conditional distribution of PRPB and is less sensitive to extreme values than ordinary least squares (OLS) regression (77).

As summarized in Table 5, both methods yielded results consistent with the baseline model across spatial dimensions. Optimism-driving factors, including per capita GDP, residents’ age, and local household registration, retained significant positive effects, indicating stable promotion of optimism risk perception. Similarly, pessimism-driving factors such as urban population density, female gender, and education level consistently reinforced pessimism bias (or were non-significant in physical space for density). These robust and congruent outcomes across methods confirm the stability of the baseline estimates and strengthen the academic credibility of the conclusions regarding key influencing factors of PRPB.

**Table 5:** Results of robustness tests for benchmark regression.

Public risk perception bias	2% Winsorization				Quantile Regression			
	(1)	(2)	(3)	(4)	(1)	(2)	(3)	(4)
	Overall level	Physical space	Social space	Cyber space	Overall level	Physical space	Social space	Cyber space
*Per Capita* GDP	0.071^***^	0.063^***^	0.077^***^	0.075^***^	0.071^***^	0.090^***^	0.070^***^	0.080^***^
	(0.008)	(0.010)	(0.009)	(0.011)	(0.011)	(0.016)	(0.013)	(0.017)
Population density	−0.007^**^	0.003	−0.008^***^	−0.017^***^	−0.008^*^	−0.001	−0.014^***^	−0.020^***^
	(0.003)	(0.003)	(0.003)	(0.004)	(0.004)	(0.005)	(0.004)	(0.006)
Gender	−0.050^***^	−0.038^***^	−0.064^***^	−0.046^***^	−0.061^***^	−0.052^***^	−0.081^***^	−0.055^***^
	(0.004)	(0.005)	(0.005)	(0.006)	(0.006)	(0.008)	(0.007)	(0.009)
Age	0.010^***^	0.006^*^	0.017^***^	0.007^*^	0.011^***^	0.012^**^	0.021^***^	0.005
	(0.003)	(0.003)	(0.003)	(0.004)	(0.004)	(0.005)	(0.004)	(0.006)
Hukou type	0.011^***^	0.011^***^	0.014^***^	0.007^**^	0.012^***^	0.020^***^	0.019^***^	0.011^**^
	(0.002)	(0.002)	(0.002)	(0.003)	(0.003)	(0.004)	(0.003)	(0.004)
Education level	−0.012^***^	−0.008^**^	−0.004	−0.029^***^	−0.018^***^	−0.013^**^	−0.008^*^	−0.043^***^
	(0.003)	(0.003)	(0.003)	(0.004)	(0.004)	(0.006)	(0.004)	(0.006)
Income level	0.002	0.002	0.004^**^	−0.001	0.007^***^	0.007^**^	0.007^***^	0.001
	(0.002)	(0.002)	(0.002)	(0.002)	(0.002)	(0.003)	(0.003)	(0.003)
Constant	−0.780^***^	−0.777^***^	−0.696^***^	−0.941^***^	−0.752^***^	−1.011^***^	−0.534^***^	−0.911^***^
	(0.088)	(0.105)	(0.094)	(0.118)	(0.122)	(0.165)	(0.134)	(0.177)
*N*	9,146	9,146	9,146	9,146	9,146	9,146	9,146	9,146

^***^, ^**^, and ^*^ represent significance levels of 1, 5, and 10%, respectively.

#### 3.3.2 Robustness test for mediating effects

To validate the reliability of the multi-level mediation pathways identified in Section 3.2.2, this study employed the Bootstrap method with 5,000 resamples, adopting 95% confidence intervals (CIs) to test significance (78). The results consistently confirmed the baseline mediation findings across all systemic levels.

In the microsystem, psychological stress mediates two pathways with modest but significant indirect effects: gender to PRPB (*β* = −0.00053871, 95% CI [−0.0011383, −0.0001434], 0.99% of total effect) and hukou type to PRPB (*β* = 0.00014551, 95% CI [0.0000173, 0.0003763], 1.41% of total effect).

The mesosystem analysis revealed social capital as the strongest mediator, particularly for population density (*β* = 0.00143333, 95% CI [0.0007128, 0.0022245], 18.04% of total effect) and hukou type (*β* = 0.00130323, 95% CI [0.0007508, 0.0019336], 12.65% of total effect), while education level showed an adverse mediating effect (*β* = −0.00091411, 95% CI [−0.0016644, −0.0002074], 5.34% of total effect), underscoring social capital’s pivotal role.

Media rationality in the exosystem exhibited relatively weaker but consistent mediation across all three predictors: population density (*β* = 0.00044806, 95% CI [0.0001909, 0.0008573], 5.64% of total effect), hukou type (*β* = 0.00041049, 95% CI [0.0001867, 0.000755], 3.99% of total effect), and education level (*β* = 0.00095483, 95% CI [0.0005134, 0.0015311], 5.58% of total effect).

At the macrosystem level, government trust distinctly mediated per capita GDP (*β* = 0.00377424, 95% CI [0.0016996, 0.0059135], 5.69% of total effect) and education level (*β* = −0.00102322, 95% CI [−0.0018602, −0.0002161], 5.98% of total effect), demonstrating its unique regulatory function.

These robust results fully corroborate the baseline mediation regression, confirming the stability of multi-level influencing mechanisms in shaping PRPB. The findings not only substantiate the research hypotheses but also provide empirically grounded insights into the formation of mechanisms of PRPB.

## 4 Conclusion

### 4.1 Main conclusions

Guided by the Tri-Spatial Framework, this study developed an Extended Tri-Spatial Prism Model to investigate PRPB across 31 major Chinese cities (2017–2023). A novel measurement system for PRPB was developed using the Delphi technique for indicator selection and the entropy method for objective weighting. Multi-source data fusion was employed to operationalize the index, while GIS-based spatial analysis and time-series modeling revealed its spatiotemporal dynamics. Furthermore, multiple regression and mediation analyses identified multi-level mechanisms through which psychological stress, social capital, media rationality, and government trust are correlated with PRPB. The principal findings are as follows:

First, overall public risk perception demonstrates systematic pessimism bias (*M* = −0.132), yet significant divergence exists across spatial dimensions: pronounced pessimism bias prevails in physical space (*M* = −0.231) and cyber space (*M* = −0.215), while social space approaches cognitive equilibrium with mild optimism (*M* = 0.015). The tri-spatial divergence of PRPB reveals the complexity of risk cognition in modern society.

Second, temporal analysis from 2017 to 2023 reveals a distinct “V-shaped” trajectory in PRPB. Pessimism intensified markedly during 2017–2020 in both physical space (from −0.069 to −0.364) and social space (from 0.025 to −0.061), reaching its nadir in 2020 under the impact of the COVID-19 pandemic, followed by a gradual post-2020 recovery. In contrast, cyber space exhibited sustained improvement, with pessimism bias decreasing at an annual rate of 27.6% since 2018; however, its persistent negative value by 2023 underscores the considerable challenge in reversing deeply embedded cognitive biases within the cyber space. This pattern highlights both the acute disruption caused by major public health crises and the incremental effectiveness of long-term risk governance policies.

Third, spatial analysis identifies a pre-pandemic north–south divergence, with northern cities (e.g., Harbin: 0.283) displaying greater optimism and southern cities (e.g., Hangzhou: −0.308) showing stronger pessimism. Although the COVID-19 pandemic induced a nationwide pessimistic shift, post-pandemic recovery diverged significantly: cities such as Shanghai (0.119) and Shijiazhuang (0.232) rebounded to optimism, while others like Zhengzhou (−0.429) retained heightened pessimism. This spatial restructuring underscores the synergistic influence of regional economic resilience, governance efficacy, and differentiated policy support.

Fourth, mechanistic analysis reveals multi-level pathways through which individual and city-level factors are correlated with PRPB. Direct effects indicate that per capita GDP (*β* = 0.072), age (*β* = 0.010), and local hukou status, (*β* = 0.011) show a positive correlation with PRPB, whereas population density (*β* = −0.007), female gender (*β* = −0.051), and education level (*β* = −0.012) show a negative correlation with PRPB. Furthermore, mediation pathways confirm that psychological stress (*β* = −0.028) amplifies pessimism, while social capital (*β* = 0.125), media rationality (*β* = 0.047), and government trust (*β* = 0.136) facilitate optimistic shifts. These findings provide a systematic theoretical basis for optimizing risk communication strategies.

### 4.2 Policy implications

Based on the empirical findings, this study proposes the following policy recommendations to optimize public risk communication and enhance urban safety governance within the tri-spatial framework:

First, adopt spatially differentiated public risk communication strategies to correct PRPB and foster rational public risk perception. In physical and cyber spaces where pessimism bias is prominent, governments should collaborate with research institutions to transform complex risk data into publicly accessible visualizations. To this end, relevant authorities need to establish rapid-response mechanisms to refute misinformation, mitigate the media’s amplification of extreme events, and disseminate governance outcomes. In the social space characterized by relative cognitive equilibrium, policies should prioritize strengthening community dialogue and information sharing, leveraging “collective intelligence” to enhance the public’s rational risk perception.

Second, implement targeted interventions for risk-sensitive groups to boost societal resilience. Tailored communication strategies should be designed for populations with heightened risk perception—such as women, highly educated individuals, and rural migrant residents—to alleviate their pessimism bias. Concurrently, promote media literacy programs to enhance the public’s ability to discern information credibility and resist misinformation. Governments should also strengthen transparency in risk information disclosure to foster public trust, while supporting community mutual-aid initiatives to expand participation in social capital. These integrated measures can systematically mitigate cognitive biases at both individual and collective levels.

Third, advance regionally coordinated and context-adaptive risk governance. Local governments should develop risk management strategies aligned with their specific socio-economic conditions, cultural contexts, and risk profiles. Establish a cross-regional knowledge-sharing platform to systematically document and disseminate governance experiences from cities with well-calibrated public risk perception (e.g., Shanghai and Shijiazhuang), serving as benchmarks for cities with high perception bias. Furthermore, target policy support and evidence-based interventions in areas with significant discrepancies between subjective and objective risk levels. Through rigorous evidence-based assessment and optimized resource reallocation, these measures can narrow the risk perception gap and facilitate the development of a rational, place-based risk governance paradigm.

### 4.3 Research limitations and future directions

This study has limitations that point to avenues for further exploration, particularly in expanding the generalizability of the Extended Tri-Spatial Prism Model. First, its sample is confined to 31 major Chinese cities, excluding small- and medium-sized cities and rural areas, which may limit the applicability of findings to non-urban or less developed regions. Second, the single-country design restricts analysis of how cultural values (e.g., individualism vs. collectivism) and institutional environments (e.g., decentralized vs. centralized governance) shape PRPB. Third, in measuring actual objective risk, the use of Baidu Index data may be susceptible to the Social Amplification of Risk Framework (SARF), potentially leading to the overestimation or misrepresentation of actual objective risk levels. Conversely, the use of criminal case statistics may result in underestimation due to systematic reporting gaps. These constitute a fundamental methodological limitation that can be mitigated but not resolved through standardization.

To address these gaps, future research should pursue two pivotal directions: (1) Expand the geographical scope of sampling through stratified random sampling designs incorporating small and medium-sized cities and rural areas, thereby investigating the differential correlation between urban–rural heterogeneity and PRPB. (2) Conduct cross-national comparative research in diverse cultural contexts (e.g., individualistic vs. collectivist societies). Such studies should focus on the interactions among cultural dimensions, governance models, and PRPB to develop a more comprehensive analytical framework. (3) The objective risk assessment system could be further optimized through the systematic integration of additional authoritative multi-source data, thereby facilitating a more precise characterization of actual objective risk levels.

## Data Availability

The raw data supporting the conclusions of this article will be made available by the authors, without undue reservation.
